# Sonication-Free Dispersion of Single-Walled Carbon Nanotubes for High-Sorption-Capacity Aerogel Fabrication

**DOI:** 10.3390/molecules27217657

**Published:** 2022-11-07

**Authors:** Dong Li, Liantao Xin, Bocheng Yang, Zizheng Chen, Qianru Wu, Fangqian Han, Shulan Hao, Lihu Feng, Xiaoyu Wang, Shiying Wang, Lei Wang, Maoshuai He

**Affiliations:** 1 College of Chemistry and Molecular Engineering, Qingdao University of Science and Technology, Qingdao 266042, China; 2 Shandong Engineering Research Center for Marine Environment Corrosion and Safety Protection, College of Environment and Safety Engineering, Qingdao University of Science and Technology, Qingdao 266042, China

**Keywords:** sonication-free, polyvinylpyrrolidone wrapping, aqueous dispersion, carbon nanotubide, aerogel

## Abstract

Homogenously dispersing single-walled carbon nanotubes (SWNTs) in solvents has been one critical step towards exploiting their exceptional properties in high-performance components. However, the solubility of SWNTs is severely limited by the inert tube surfaces and strong tube-tube van der Waals attractions. Starting with carbon nanotubides, i.e., negatively charged SWNTs reduced by alkali metals, we herein propose a sonication-free approach to prepare an aqueous dispersion of SWNTs. The approach combines the spontaneous dissolution of nanotubides in polar aprotic solvents with polyvinylpyrrolidone wrapping and dialysis in deionized H_2_O, which results in well-dispersed, neutralized SWNTs. The gelation of concentrated SWNT dispersion leads to the formation of hydrogels, which is subsequently transformed into SWNT aerogels through lyophilization. The prepared SWNT aerogels exhibit high-mass-sorption capacities for organic solvent absorption, paving the way towards harvesting the extraordinary properties of SWNTs.

## 1. Introduction

The dispersion of single-walled carbon nanotubes (SWNTs) is a first critical step towards harnessing the superior optoelectronic and mechanical properties of individual SWNTs [1,2,3,4,5,6,7,8]. Using either non-covalent or covalent functionalization methods, great efforts have been made to prepare dispersed SWNT derivatives since the 1990s [9,10,11]. In 2002, O’Connell et al. used the shearing forces of ultrasonication to debundle and stabilize SWNTs with surfactants [10]. However, the harsh sonication applied during the processing severely deteriorated their pristine structures and shortened most of the nanotubes [12,13]. In contrast, chemical modification could maintain the SWNTs’ structural integrity; nevertheless, the covalent functionalization of nanotube sidewalls causes the optical properties to permanently vanish. Consequently, dispersing SWNTs without shortening or chemical functionalization remains an obstacle to benefiting from their cutting-edge applications.

Nanotubides represent a class of alkali metal-reduced carbon nanotubes, which consist of negatively charged carbon nanotubes and surrounding counterions. Pioneer work for preparing nanotubides by doping bulk SWNTs with potassium was reported by Smalley’s group in 1997 [14]. Later, Petit et al. applied metallic lithium and polyaromatic molecules in tetrahydrofuran (THF) to reduce SWNT films [15]. By refining Petit’s method, Penicaud et al. synthesized uniformly charged nanotubides [16], which can spontaneously dissolve in a series of aprotic organic solvents with a high SWNT solubility. For example, nanotubides dissolved in dimethyl sulfoxide (DMSO) can reach a concentration of up to 9.4 mg/g [17], suitable for manufacturing macroscopic SWNT materials such as SWNT aerogels and fibers [18]. Moreover, compared with the sonication process, the spontaneous dispersion of nanotubides in organic solvents can avoid the shortening of SWNTs [19]. Nonetheless, the dispersion is unstable in air and the optical properties of SWNTs are suppressed by electron filling, which hinders the potential applications of SWNT dispersion.

In addition to SWNT dispersion, assembling SWNTs into functional materials, such as aerogels, is also of great interest. Owing to the low density, high specific surface area, and hydrophobic surfaces [2], the SWNT aerogels have shown great potential for organic solvent sorption, which is anticipated to solve the environmental problems caused by frequent oil or organic solvent spill accidents [20,21]. In 2007, Yodh et al. converted the SWNT aqueous dispersion into aerogels by freeze-drying and supercritical drying [22]. Due to the strain-induced intertube sliding [23], such SWNT networks are fragile under compression. To reinforce SWNT aerogels, some functional materials such as polydimethylsiloxane [24,25], epoxy resin [26], graphene [27], and oxide were applied [28,29]. These functional materials could effectively enhance the stretching stability of SWNT aerogels, but inevitably cause a dramatic decrease in specific surface area and increase the aerogels’ density, accounting for the relatively low sorption capacities of organic solvents.

With the aim of overcoming the instability of charged SWNT dispersion in atmospheric environments and restore their intrinsic optical properties, we propose to combine the spontaneous dissolution of nanotubides in organic solvents with polyvinylpyrrolidone (PVP) wrapping and dialysis to preparing neutralized SWNT dispersion. The recovery of SWNT optical properties will be investigated by Raman spectroscopy and UV–vis–NIR absorption spectroscopy. To explore the potential applications, the SWNT dispersion is subsequently concentrated and transformed into hydrogels through water evaporation. After freeze drying and heat treatment, SWNT aerogels assembled by the recovered tubes are obtained. Finally, the capabilities of SWNT aerogels in absorbing different organic solvents are systematically investigated.

## 2. Results

### 2.1. Characterizations of SWNT Dispersions

**Figure 1:**
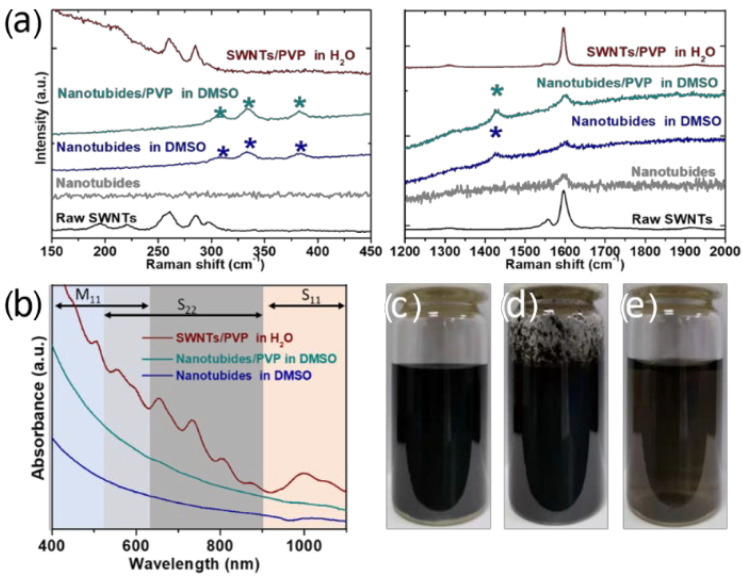
(**a**) Raman spectra of SWNTs at different stages, taken with a 633 nm laser excitation. The bands marked with * come from DMSO. From the bottom to the top are the raw SWNTs, nanotubides, nanotubides in DMSO, nanotubides/PVP in DMSO, and SWNTs/PVP in water. (**b**) UV–vis–NIR absorption spectra of SWNTs/PVP in H_2_O, nanotubides/PVP in DMSO, and nanotubides in DMSO. Photographs of nanotubide dispersion in DMSO before (**c**) and after (**d**) being exposed to air. (**e**) Photograph of neutralized nanotubide/PVP aqueous dispersion.

Raw SWNT soots were applied to prepare the nanotubides. The bottom spectrum of Figure 1a presents the Raman spectrum of raw HiPco SWNTs, which exhibits radial breathing modes (RBMs), the D mode and G mode, corresponding to SWNT radial expansions–contractions, structural defects, and the planar vibrations of carbon atoms, respectively. Grounded in the frequencies of RBM bands, the diameters of SWNTs are estimated in the range of 0.7–1.2 nm, in agreement with previous reports [30]. Reacting HiPco SWNTs with the THF solution containing Na^+^ Naphtalene^-^ in an Ar atmosphere led to the formation of nanotubides [16]. During the reaction, raw SWNTs are reduced by metallic sodium. Due to the occupation of conduction bands by alkali metal doping, the disappearance of RBM bands is accompanied by the weakening of the G mode in the Raman spectrum (Figure 1a). After filtration and drying, the nanotubides were readily dissolved in DMSO, and hardly any visible agglomerates were observed (Figure 1c). Thanks to the Coulomb-driven exfoliation forces among negatively charged SWNTs, the nanotube polyelectrolytes in polar aprotic solvents are at thermodynamic equilibrium, and can thus spontaneously dissolve in DMSO and be indefinitely stable in an inert atmosphere [16]. Absorption bands corresponding to transitions between the mirror spikes of density of states are suppressed (Figure 1b), indicating that the initially empty states are filled. Unfortunately, the nanotubide-DMSO solution is unstable when exposed to air or H_2_O, causing severe aggregation. Upon air exposure, nanotubides reduced the oxygen to the superoxide anion, which eventually yields the hydroxide anion [31], whereas nanotubides lose their charge and turn to neutral SWNTs because of the extremely low reduction potential [32]. Due to the lack of repulsive forces between neutralized SWNTs, the van der Waals interactions tend to aggregate them again (Figure 1d). In order to maintain the high dispersion degree of nanotubides after oxidation, the PVP molecules were first added into the nanotubide–DMSO solution to wrap the nanotubides and a subsequent dialysis in deionized water was conducted to discharge the nanotubides and replace the DMSO. Compared with the nanotubides, the nanotubides/PVP show a similar oxidation behavior. After the dialysis of nanotubides/PVP in air, the appearance of characteristic peaks in the Raman spectra and UV–vis–NIR absorption spectrum is correlated with the allowed electronic/optical transitions in neutral SWNTs, indicative of the wrapping of PVP not influencing the oxidation process of nanotubides (Figure 1a,b). Moreover, minor functionalization was found in oxidized nanotubide/PVP by infrared spectra, which is consistent with the oxidized nanotubides (Supplementary Materials Figure S1). However, in contrast with the unstable dispersion state of nanotubide–DMSO in air, the nanotubide/PVP-DMSO could maintain its good dispersion state even after undergoing dialysis in the atmospheric environment (Figure 1e). This is attributed to the wrapping of PVP, which could disrupt the tube–tube van der Waals interaction and hinder the aggregation of discharged nanotubides. The phenomena highlight the importance of using PVP to wrap nanotubides. Figure 1a presents the Raman spectrum of nanotubide/PVP dispersion in DMSO. Similar to the raw nanotubides, the nanotubide/PVP dispersion demonstrates the absence of RBM bands and a globally much weaker signal. The Raman characterization results suggest that apart from non-covalent wrapping, PVP does not react with nanotubides in DMSO. It is noted that when other surfactants or polymers, such as sodium dodecyl sulfate, cetrimonium bromide, or poly(vinyl alcohol) instead of PVP are added, instant aggregation occurs because of the charge neutralization of nanotubides by the cations. Similar to the nanotubide–DMSO dispersion, hardly any peak was observed in the absorption spectrum (Figure 1b). The Raman spectrum of the SWNT/PVP dispersion after dialysis exhibits the characteristic features of neutralized SWNTs (Figure 1a). Particularly, the narrow G mode indicates the presence of large quantities of individual SWNTs in dispersion [33]. In addition, the absorption features can also be applied to evaluate the dispersion degree of SWNTs [10]. If the SWNTs are in bundle states, exciton energy transfer would decrease the lifetime of excited excitonic states, thus broadening the widths of the absorption peaks. In contrast, individual SWNTs display a series of well-resolved fine features because of their sharp excitonic transitions. Note that the absorption spectrum of sonication-free SWNT/PVP dispersion exhibits sharp characteristic peaks, indicating that the SWNTs were well dispersed.

**Figure 2:**
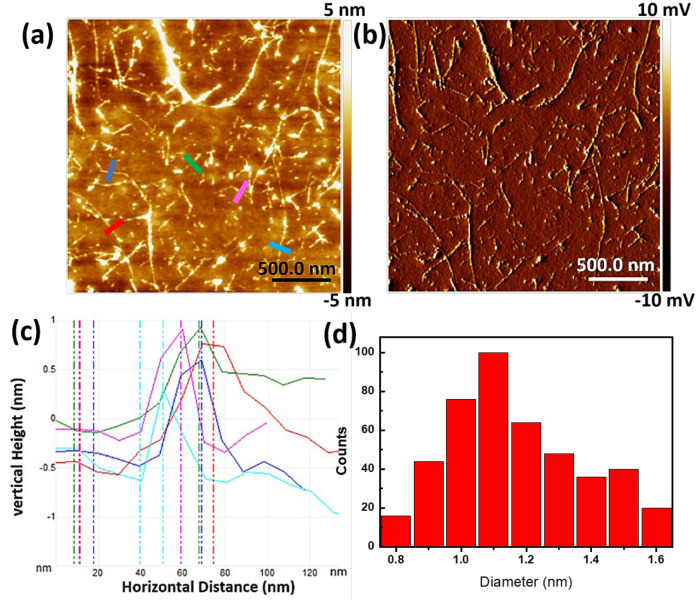
Atomic force microscopy (**a**) height image and (**b**) amplitude error image of dispersed SWNTs on a Si substrate. (**c**) Height analysis of the SWNTs marked by the colored line in image (**a**). (**d**) Diameter distribution of dispersed SWNTs.

Atomic force microscopy characterizations were performed to further verify the dispersion of SWNTs cast onto a bare Si substrate (Figure 2a). The surfaces of dried SWNTs are not very smooth, suggesting the wrapping of SWNTs by PVP via a thermodynamic wrapping configuration [34]. Height analysis from the bare sections of SWNTs gives a diameter distribution ranging from 0.8 to 1.6 nm (Figure 2c,d), close to the diameters of starting HiPco SWNTs. The results also confirm that the dispersion mainly contains individual tubes or small bundles.

### 2.2. Preparation and Characterizations of SWNT Aerogels

**Figure 3:**
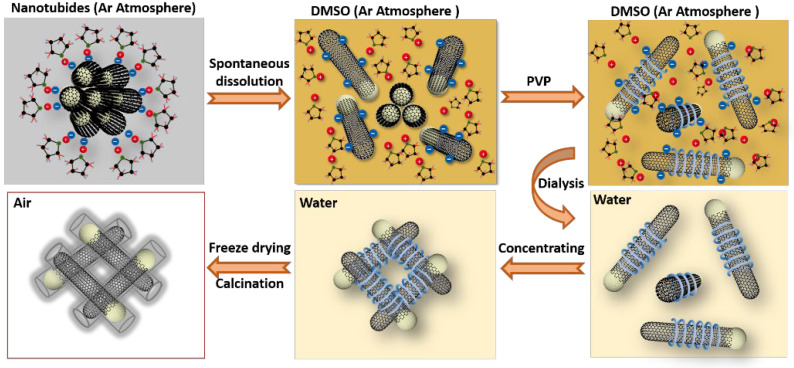
Schematic illustration of the procedure for SWNT dispersion and aerogel preparation.

**Figure 4:**
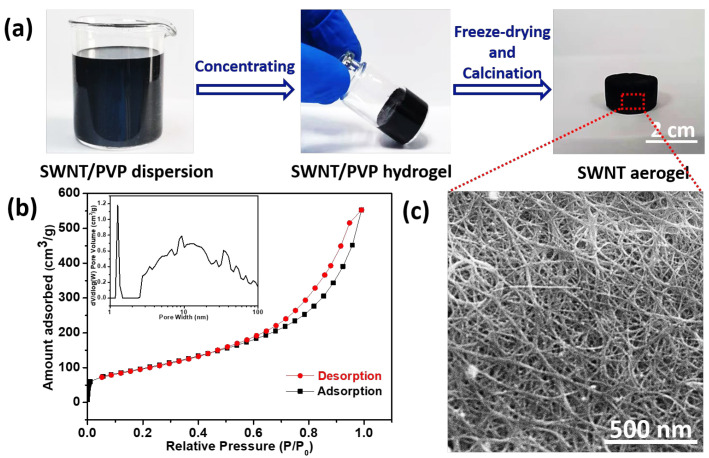
(**a**) Photographs of the SWNT/PVP dispersion, SWNT/PVP hydrogel, and SWNT aerogel. (**b**) N_2_ adsorption–desorption isotherm of SWNT aerogels. Inset is the pore size distribution profile obtained from the adsorption branch. (**c**) SEM image of SWNT aerogels.

The abovementioned SWNT dispersion strategy is also applicable to Tuball SWNTs, which can be supplied in a large quantity. The illustration for the transformation from SWNT dispersion to aerogels is shown in Figure 3. The SWNT hydrogels were first prepared by the concentration of SWNT dispersion. After the subsequent freeze-drying and heat treatment, the SWNT aerogels are obtained. Figure 4a presents the optical photograph of prepared SWNT/PVP dispersion with an estimated SWNT concentration of 1.18 mg/mL. By controlling the water evaporation, the concentration of SWNTs was increased to 6 mg/mL. The concentrated SWNT suspension was opaque and very viscous, the standing of which for 12 h led to the formation of SWNT/PVP hydrogels (Figure 4a). This phenomenon can be explained by the topological entanglements of SWNTs which take place when the SWNT concentration is higher than a threshold value. After lyophilization, the hydrogels were transformed into aerogels without significant volume shrinkage, and the PVP molecules wrapped on SWNT surfaces could be decomposed by calcination in Ar. The density of SWNT aerogels is directly correlated with the concentration of SWNTs in SWNT/PVP hydrogels. As the dispersed SWNTs exhibit a high aspect ratio, gelation readily occurs at an SWNT threshold concentration of 6 mg/mL, resulting in SWNT aerogels with a similar density after lyophilization. The determined BET specific surface area is 357 m^2^/g with a pore volume of up to 0.67 cm^3^/g at P/P_0_ = 0.95 (Figure 4b), and the pore size distribution profile (Inset of Figure 4b) provides evidence of the existence of hierarchical pores in aerogels [35,36]. Scanning electron microscopy (SEM) (Figure 4c) and transmission electron microscopy (TEM) characterizations further confirm the assembly of SWNTs in porous aerogels (Supplementary Materials Figure S2).

### 2.3. Applications of SWNT Aerogels for Organic Solvent and Oil Absorption

**Figure 5:**
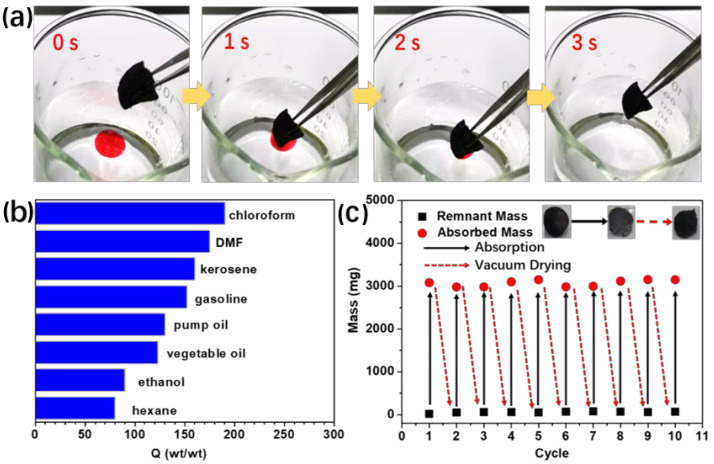
(**a**) Absorption of pump oil (colored with Sudan III for clear presentation) by SWNT aerogels within 3 s. (**b**) Absorption capacities of SWNT aerogels for various organic solvents. (**c**) Reusability of SWNT aerogels for kerosene absorption.

**Figure 6:**
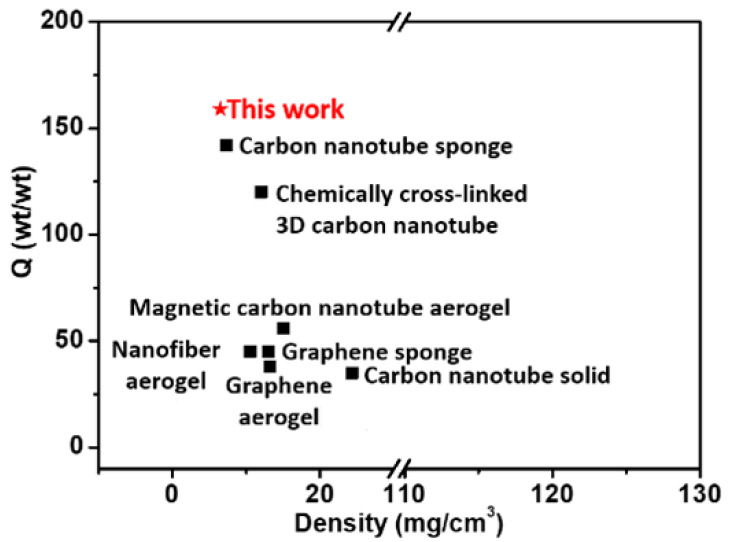
The comparison of kerosene sorption capacities of SWNT aerogels and previously reported sorbents. Carbon nanotube sponge [20]; Chemically cross-linked 3D carbon nanotube [37]; Magnetic carbon nanotube aerogel [21]; Nanofiber aerogel [36]; Graphene sponge [38]; Graphene aerogel [39]; Carbon nanotube solid [40].

The contact angle of water with SWNT aerogels is more than 120° (Supplementary Materials Figure S3), correlated with the hydrophobicity of the SWNT surface and the porous structures. Consequently, SWNT aerogels were tested for organic solvent absorption. Figure 5a and Supplementary Materials Video S1 show the process of a piece of aerogel absorbing the pump oil dyed with Sudan III, which can be completed in 3 s, leaving clean deionized water. Figure 5b presents the absorption capacities of SWNT aerogels to various organic solvents, including chloroform, dimethylformamide (DMF), kerosene, gasoline, pump oil, vegetable oil, ethanol, and hexane. The aerogels exhibit high absorption capacities for all organic solvents, ranging from 80 to 190 g/g (Figure 5b). The absorbed kerosene in the aerogels can be removed by vacuum drying and the aerogels could be reused. After kerosene absorption, the aerogel mass increased from 19 mg to 3122 mg while the volume of aerogel only slightly increased (approximately 10%, Figure 5c). After conducting the absorption and desorption of kerosene for 10 times, no obvious decrease in absorption capacities was observed, and the aerogel almost recovered its original shape. Figure 6 compares the kerosene sorption capacities of SWNT aerogels with those of other sorbents [20,21,36,37,38,39,40]. The SWNT aerogels exhibit a sorption capacity of nearly 160 g/g, which is higher than that of carbon sorbents. Such a higher sorption capacity could be related to the low density (~6 mg/cm^3^) and the large specific surface area (~357 m^2^/g), providing a large volume at unit mass to absorb and store kerosene. The stable recyclability and high absorption capacities render the SWNT aerogels a promising candidate for applications in environmental protection and sewage disposal.

## 3. Materials and Methods

### 3.1. Preparation of Nanotubides

Two types of commercial SWNTs, HiPco SWNTs (Batch No. HR27-075) and Tuball SWNTs (Batch No. 521), were used in the experiments. The procedure for preparing nanotubides was described in a previous report and performed in a glove box (Mikrouna, Universal 2440/750/900) filled with Ar (99.999%) [16]. Briefly, the naphthalene with a mass of 0.384 g (Shanghai, China, Macklin, 99%) was first dissolved in 100 mL THF (Shanghai, China, Macklin, 99.5%, water ≤ 50 ppm) and reacted with 0.07 g sodium (Shanghai, China, Sinopharm Chemical Reagent limited corporation, AR) to form a solution of Na^+^Naphtalene^-^. Under magnetic stirring at a speed of 400 rpm/min, the reaction was conducted at room temperature for 24 h. Then, 0.2 g SWNTs were added into the resulting dark green solution and stirred for another 12 h at room temperature. After filtration over a 0.45 µm PVDF membrane, the obtained nanotubides were rinsed with THF and dried in vacuum at room temperature.

### 3.2. Preparation of Aqueous Dispersion of SWNTs

A total of 20 mg nanotubides was dissolved in 20 mL DMSO (Shanghai, China, Macklin, 99.7% water ≤ 50 ppm) with magnetic stirring (400 rpm/min), which was subsequently poured over 20 mg PVP (Mw = 90,000). After PVP dissolution, the solution was transferred out of the glove box and exposed to air. Dialysis was carried out by using a dialysis bag (MWCO = 500) in deionized water (Milli-Q, 18.25 MΩ cm). Finally, the PVP wrapped SWNTs were centrifuged (11,000 g) for 30 min to remove the SWNT bundles and only the supernatant was collected. To estimate the concentration of SWNTs, 50 mL as prepared SWNT dispersion was freeze-dried and annealed at 700 °C in Ar for 1 h to decompose the PVP. The residual black powders were weighed to be 59.2 mg, and the calculated SWNT concentration was 1.18 mg/mL.

### 3.3. Preparation of SWNT Hydrogels and Aerogels

The concentration of the SWNT dispersion was first concentrated to approximately 6 mg/mL by evaporating H_2_O at 90 °C. After removing the bubbles by degassing, the 3 mL concentrated SWNT dispersion was poured into a cylindrical mold with a diameter of 20 mm and stood for 12 h to form hydrogels. SWNT aerogels were fabricated by the freeze drying of the hydrogels, followed by annealing in Ar atmosphere at 700 °C for 1 h to decompose the wrapped PVP.

### 3.4. Characterizations of SWNT Dispersions and Aerogels

The raw SWNTs and SWNT dispersions were evaluated by a Raman spectroscope (UK, Renishaw, inVia confocal) with excitation wavelengths of 532 nm and 633 nm. Fourier transform infrared spectroscopy (Germany, Bruker, VERTEX 70) was employed to analyze the functional groups on SWNT sidewalls. Atomic force microscopy (USA, Bruker, Multimode 8) was applied to characterize the SWNTs dispersed onto a Si substrate. The optical absorption of SWNT dispersion was surveyed with a UV–vis–NIR spectrophotometer (Japan, Shimadzu, UV-3600). The microscopic morphologies of aerogels were investigated by scanning electron microscopy (Japan, Hitachi, Regulus 8100) and transmission electron microscopy (Japan, JOEL, 2100F). The nitrogen adsorption–desorption measurements of aerogels were performed on a surface area and porosity analyzer (USA, Micromeritics, ASAP2460-2).

### 3.5. Organic Solvent Sorption of SWNT Aerogels

Cylindrical SWNT aerogels with a diameter of 20 mm and a height of 10 mm were put into 20 mL different organic solvents at room temperature. After sorption, the aerogels were lifted up to drip for 30 s prior to being weighed. The mass sorption capacities were calculated as Q = (M-M_0_)/M_0_, where M_0_ is the initial weight of SWNT aerogels and M is the weight after organic solvent sorption. To test the reusability of SWNT aerogels, kerosene absorbed in SWNT aerogels was removed by heat treatment at 120 °C in vacuum.

## 4. Conclusions

In summary, we proposed a sonication-free approach for preparing the aqueous dispersion of SWNTs, which was realized by consecutive SWNT reduction, PVP wrapping, and dialysis in deionized H_2_O. Owing to the stabilization of PVP wrapping, the neutralized nanotubides could maintain their well dispersion states in aqueous solution, which is also verified by the characteristic optical features of the obtained SWNT/PVP dispersion. Furthermore, the SWNT/PVP dispersion could be concentrated and form hydrogels at a low SWNT concentration, leading to the generation of highly porous SWNT aerogels through lyophilization, which exhibit large capacities in organic solvent sorption. This work not only paves the way towards the dispersion of pristine SWNTs, but also enhances the prospects for environmental applications of SWNT aerogels.

## Data Availability

Not applicable.

## Supplementary Materials

The following supporting information can be downloaded at: https://www.mdpi.com/article/10.3390/molecules27217657/s1, Figure S1: Infrared spectra of raw SWNTs, PVP, and SWNT/PVP dispersion.; Figure S2: TEM image of SWNT aerogels.; Figure S3: A drop of water on SWNT aerogels.; Video S1: The process of a piece of aerogel absorbing pump oil.
