# Supplementary material for: Sonication-Free Dispersion of Single-Walled Carbon Nanotubes for High-Sorption-Capacity Aerogel Fabrication
Source: Molecules. 2022 Nov 7;27(21):7657. doi: 10.3390/molecules27217657 (PMC9658345; doi:10.3390/molecules27217657)
Supplement: Supplementary file 1 [file molecules-27-07657-s001.zip › Supplementary Material.pdf]

Sonication-Free Dispersion of Single-Walled Carbon Nanotubes for High-Sorption-Capacity Aerogel Fabrication

Dong Li [1], Liantao Xin [1], Bocheng Yang [1], Zizheng Chen [1], Qianru Wu [1], Fangqian Han [1], Shulan Hao [1], Lihu Feng [1], Xiaoyu Wang [1], Shiying Wang [1,*], Lei Wang [1,2] and Maoshuai He [1,*]

[1] College of Chemistry and Molecular Engineering, Qingdao University of Science and Technology, Qingdao 266042, China
[2] Shandong Engineering Research Center for Marine Environment Corrosion and Safety Protection, College of Environment and Safety Engineering, Qingdao University of Science and Technology, Qingdao 266042, China
* Correspondence: wangshiying@qust.edu.cn (S.W.); hemaoshuai@qust.edu.cn (M.H.); Tel.: +86-53284022681(M.H.)

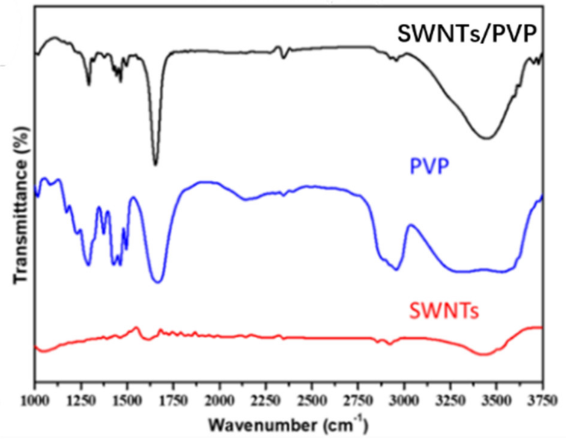

Figure S1. Infrared spectra of raw SWNTs, PVP, and SWNT/PVP dispersion.

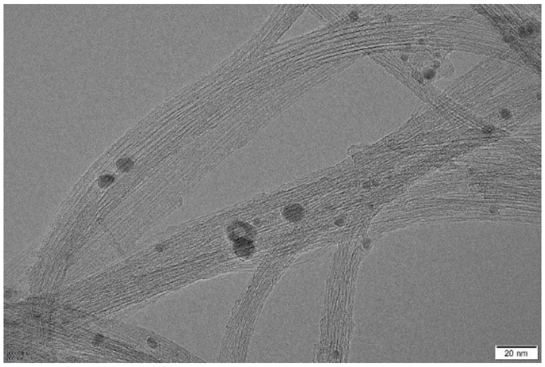

Figure S2. TEM image of SWNT aerogels.

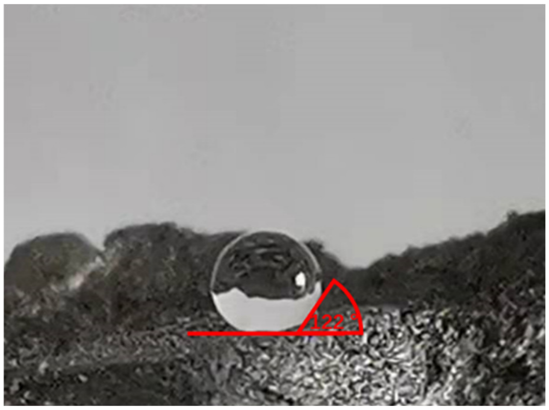

Figure S3. A drop of water on SWNT aerogels.
